# Cystic lymphangioma of pancreas: A rare case report and review of the literature

**DOI:** 10.1097/MD.0000000000011238

**Published:** 2018-07-13

**Authors:** Diyu Chen, Xiaode Feng, Zhen Lv, Xiaofeng Xu, Chaofeng Ding, Jian Wu

**Affiliations:** a Division of Hepatobiliary and Pancreatic Surgery, Department of Surgery, the First Affiliated Hospital, School of Medicine, Zhejiang University; b Key Laboratory of Combined Multi-organ Transplantation, Ministry of Public Health; c Collaborative Innovation Center for Diagnosis Treatment of Infectious Diseases, Hangzhou, Zhejiang Province, China.

**Keywords:** cyst, distal pancreatectomy, lymphangioma, neoplasm, pancreas

## Abstract

**Rationale:**

Lymphangiomas are benign lymphatic malformations that mostly occur in the neck and axillary regions. Abdominal lymphangioma is a rare type of this tumor, and pancreatic lymphangioma accounts for less than 1% of all lymphangiomas. In this report, we firstly reveal the application of ultrasound-guided puncture drainage combined with cell morphological examination for the diagnosis of pancreatic lymphangioma.

**Patient concerns:**

A 35-year-old male patient was admitted to our hospital with recurrent abdominal pain and general weakness for 1 week. From abdominal ultrasound (US) showed that a large cystic lesion occupied the abdomen, about 30.0cm×25.0cm, leading to suspicion of lymphatic cyst. Computed tomography (CT) was performed for further diagnosis and staging.

**Diagnoses:**

According to pathological findings in combination with immunohistochemical features, pancreatic lymphangioma was made.

**Interventions:**

To relieve symptoms of discomfort in the patient, distal pancreatectomy and splenectomy was carried out 1 week after the CT scan.

**Outcomes:**

The patient recovered to normal status within 19 days after surgery.

**Lessons:**

The abdominal cystic lymphangiomas are difficult to be differential diagnosed from other cystic lesions. And the origin of the tumor is also hard to be detected before operation. We should combine image and pathological examination to clarify a diagnosis. Although lymphangiomas are benign tumours, they can encroach on adjacent organs and grow to an enormous size and that, resection of these invaded organs may be required for a complete excision.

## 1 Introduction

Lymphangiomas are congenital benign hamartomas arising from the lymphatic system, and are associated with the sequestrations of lymphatic tissue during embryologic development.^[1,2]^ Lymphangiomas are mostly (∼95%) found in the neck and axillary regions of pediatric patients.^[3]^ Pancreatic cystic lymphangioma is extremely rare, accounting for <5% of pancreatic neoplasms.^[4]^ Due to the limitations of imaging technology, accessing the clinical pathological type of the pancreatic cystic lesions is difficult.

Furthermore, the symptoms of pancreatic lymphangioma are not typical of lymphangiomas in general. The symptoms can occasionally occur in response to the size and location of the tumor, and include gastrointestinal discomfort and abdominal pain.^[5]^ Our case presented with a symptomatic mass, and upon radioscopic detection, the mass was initially thought to be a lymphatic cyst. Even though lymphangiomas are considered as benign neoplasms, they can invade to adjacent organs. With different organ invaded situations, sometimes cystic resection or pancreaticoduodenectomy is necessary.^[6]^ The case described here was diagnosed as a pancreatic lymphangioma based on pathological results.

## 2 Case report

### 2.1 Patient information

A 35-year-old male presented to our out-patient department with complaints of recurrent abdominal pain and general weakness for 1 week. No history of prior surgery, trauma, or any other comorbidity existed. He had no alcohol abuse habit or familial history of pancreatic disease. The initial computed tomography (CT) scan demonstrated a large cystic lesion in the upper abdomen and the origin of the lesion could not be identified.

### 2.2 Clinical findings

**Figure 1:**
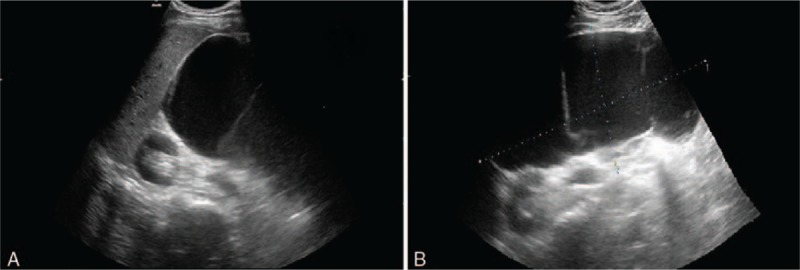
US scan detection shows a giant cystic lesion in the abdomen, with thin septa within the lesion. (A) US image of the lateral abdominal view. (B) US image of the anterior abdominal view.

**Figure 2:**
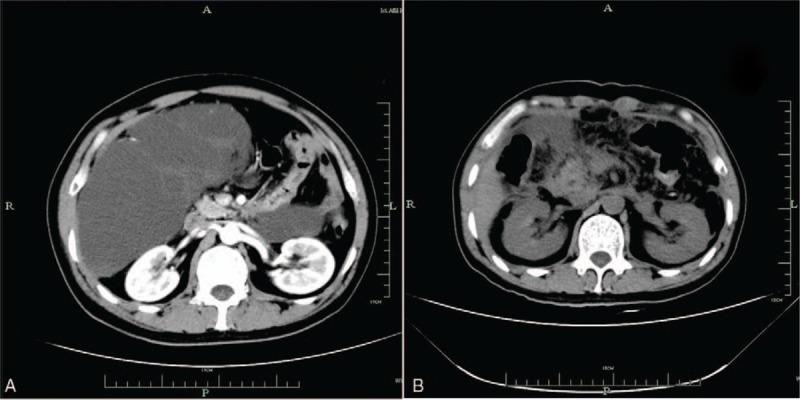
The CT scan imaging of the lymphangioma before and after the operation. (A) Before the operation, the CT scan demonstrated large cystic lesion with thin walled cavities in the abdomen. (B) The CT image after distal pancreatectomy and splenectomy.

At physical examination, an immovable abdominal mass was detected in the upper quadrant was found and the tenderness and rebound-tenderness of the whole abdomen were obvious. Initial laboratory findings revealed mild leukocytosis (10.7 × 10^9^/L), elevated neutrophil granulocytes (89% of the leukocytes) and elevated C reactive protein (CRP) (373.2 mg/L). The measured tumor markers were within the normal range. These results decreased the likelihood of a diagnosis of malignancy. His other laboratory investigations were within the normal reference ranges. Subsequently, he was admitted to the general surgery department. During his hospitalization, a CT scan and ultrasound (US) were performed again to assess the properties of abdominal lesions. The US showed that a large cystic lesion occupied the abdomen (Fig. 1). The review result of the CT scan showed a large cystic lesion of the abdominal cavity, which was considered a lymphatic cyst combined with purulent inflammation (Fig. 2A). Under the direction of the B-ultrasonic scan, we obtained 20 mL liquid from the cystic lesion through fine needle aspiration. Cell morphological examination showed a large number of lymphocytes and fewer monocytes in the cystic fluid. Then we gathered the cast-off cells, and identified the cells by immunohistochemical (IHC) staining. We observed the positivity of CD31 and D2-40 in the cast-off cells.

### 2.3 Therapeutic focus and assessment

**Figure 3:**
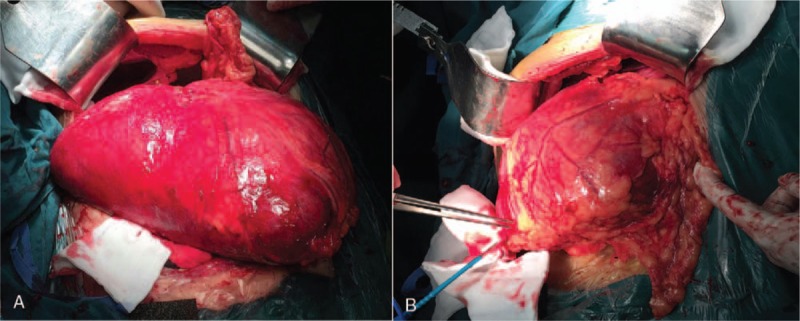
Laparotomy exploration demonstrates a large cyst and location. (A) The anterior view of the tumor during the laparotomy exploration. (B) The posterior view of the tumor during the laparotomy exploration.

**Figure 4:**
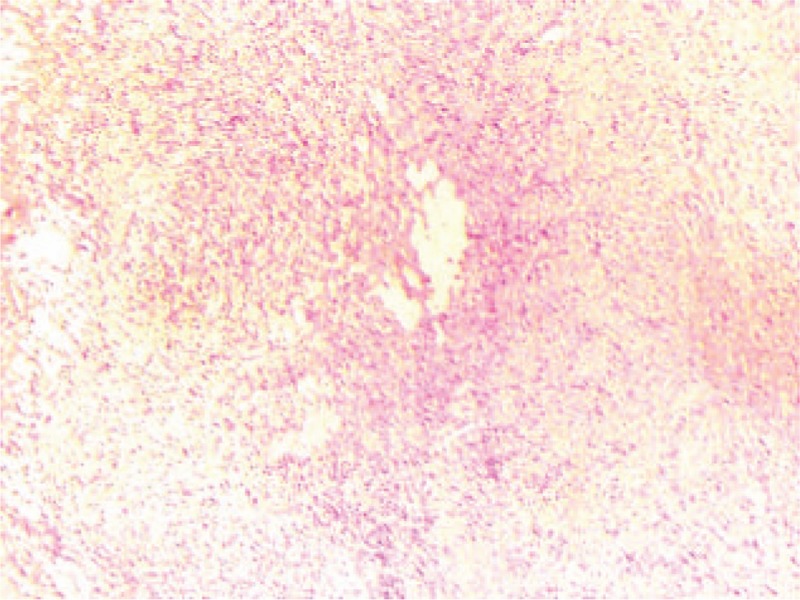
H&E staining shows that the thin wall of the pancreatic lymphangioma specimen is lined by flattened endothelial cells (original magnification × 100).

Due to the volume of tumor occupied most of abdominal cavity interspace, laparoscopic exploration was hard to perform. Therefore, the patient underwent excision laparotomy of the cyst based on clinical and radiological findings. Abdominal exploration was performed and a cystic lesion measuring approximately 40.0 cm × 30.0 cm originated from the body of pancreas and extended into the mesocolon (Fig. 3). The tumor invaded to the spleen and no invasion into vascular structures. In order to retain the pancreatic secretion function and achieve radical resection effect of the patient, distal pancreatectomy (including the cyst, the body, and tail of pancreas) and splenectomy were performed. The pathologic reports showed that, the cyst measured 22.0 cm × 15.0 cm × 5.0 cm (the out-flowing lymphatic fluid in the surgery caused decreased volume of the specimen), and the cyst had a thin membranous appearance. Histological analysis revealed the variable sizes of multiple cysts with thin walls and ectasia of the lymphatic vessels (Fig. 4). IHC stains for SMA, CD31, and D2-40 showed positivity, but CD34 staining was negative. The final pathological diagnosis was pancreatic cystic lymphangioma. After 19 days, the patient was discharged without any complications.

## 3 Discussion

### 3.1 Description of lymphangioma

Lymphangiomas are benign cystic tumors, commonly occur in the neck, axilla, and mediastinum. Three histological subtypes of the disease are predominant: cystic, capillary, and cavernous.^[7]^ However, only the cystic and cavernous types have been reported in the pancreas, and sometimes, the tumor has a mixture of these 2 histological characteristics. Until now, only a few reported cases in which the tumors from the head of the pancreas have been reported.^[8]^ As shown in Table 1, 12 of the cases had the lymphangiomas in the head of the pancreas. Four reported cases had lymphangiomas in the body, and 6 cases had lymphangiomas in the tail. The remaining cases had lymphangiomas in the head-body junction (2/20) or, in the whole pancreas (1/20).

**Table 1:**
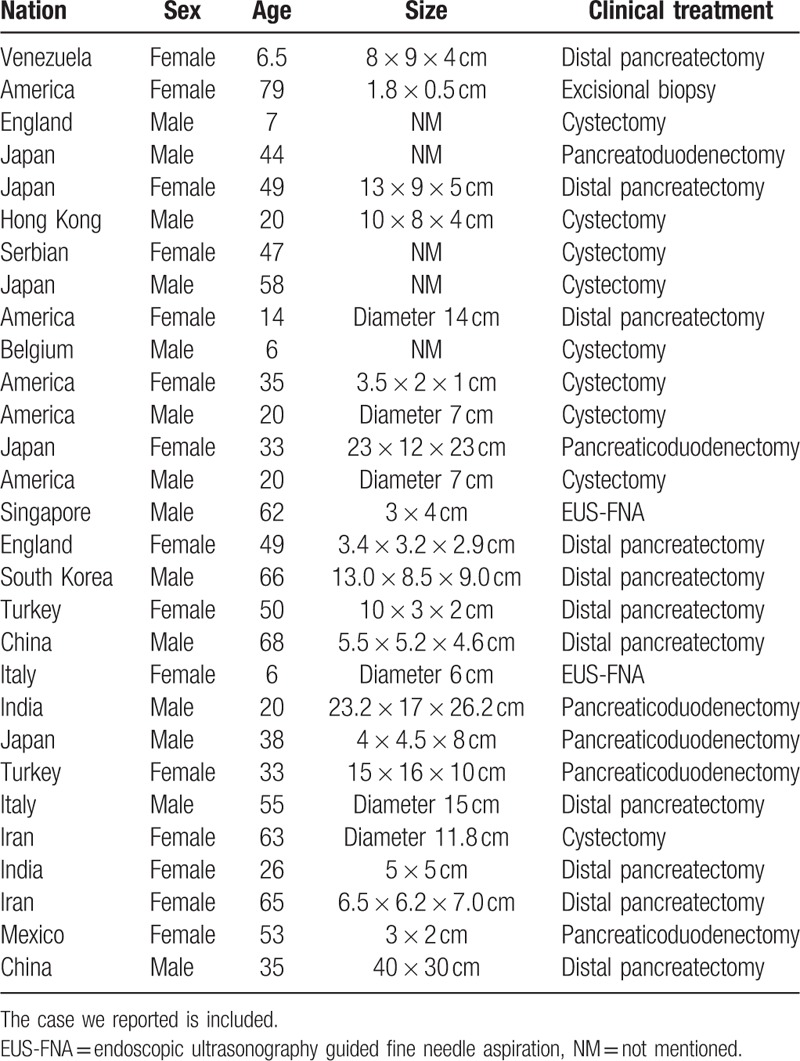
Summary information of the 29 cases reviewed in the literature.

### 3.2 Clinical findings of pancreatic lymphangioma

We reviewed 29 case reports of pancreatic lymphangioma in the literature. The patients included 15 females and 14 males, the ages ranged from 6 to 79, and the mean age was 40.3 years (Table 1). In addition, we found that most of the patients were Asians (16/29), and that Americans and Europeans comprised 7/29 and 6/29, respectively (Table 2). This observation suggests that the pathogenesis of pancreatic lymphangioma is regional.

**Table 2:**
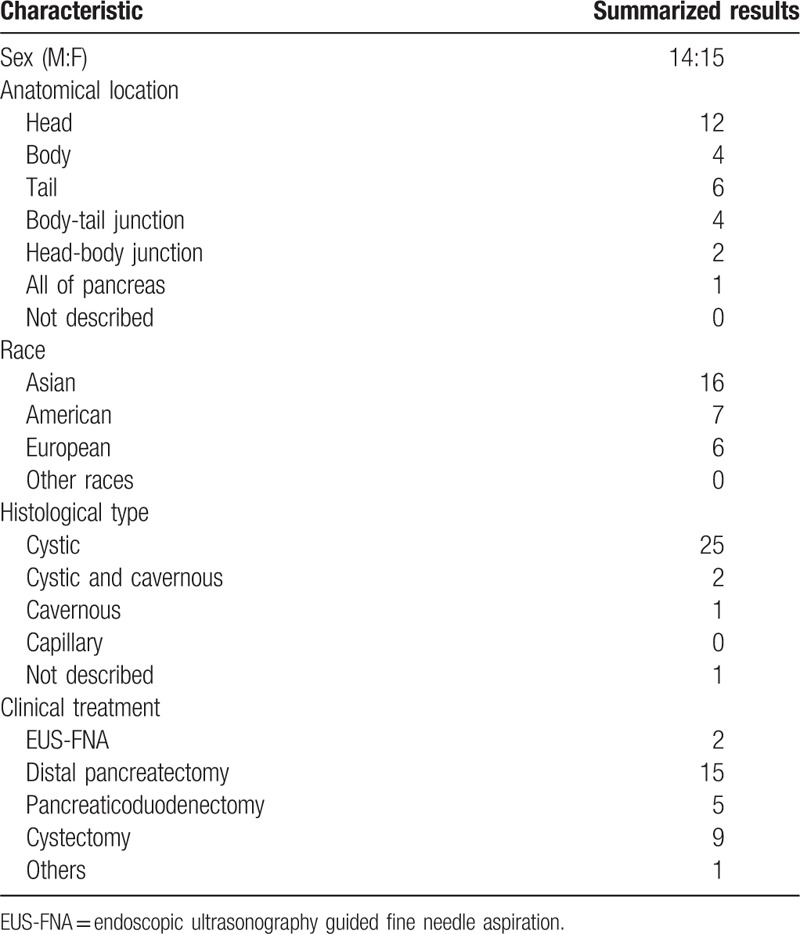
Epidemiological and pathological results of the 29 cases in the literature review.

Patients often present with various nonspecific symptoms, including abdominal pain, digestive discomfort, and a palpable mass, due to the different sizes and locations of lymphangiomas.^[9]^ However, when the tumors invade to neighboring organs, such as the adrenal glands, patients present with specific clinical manifestations, such as acute kidney injury (AKI) with rhabdomyolysis.^[10]^ Therefore, the clinical symptoms of the patients and laboratory-based detections may provide clues to identify the origin of the tumor and perform the differential diagnosis.

### 3.3 Diagnosis of pancreatic lymphangioma

Image examination is significant in the diagnosis of cystic lymphangiomas. Endoscopic ultrasound (EUS) may be a first-line procedure for the diagnosis of lymphangioma, and it will show a multilocular lesion with internal septa. In some special organs, like stomach, EUS has a high sensitivity. But for the lymphangioma located in deep viscera, its sensitivity can decrease. CT imaging is superior for lymphangioma diagnosis.^[11]^ A homogeneous cystic mass with thin walls and multiple fine intervals can be found on a CT scan, and the wall of the tumor may be enhanced after intravenous contrast-administration. Sometimes, CT scans reveal phlebolith-like calcifications of the cystic wall.^[11]^ MRI image interpretation is reportedly helpful for the differential diagnosis of cystic lymphangiomas. The cystic spaces appear hypointense on T1-weighted images and hyperintense on T2-weighted images. However, the origin of lymphangiomas is difficult to determine by imaging.^[5]^

At present, IHC staining is an accurate way to diagnose cystic lymphangioma of the pancreas. The tumors are formed by irregular dilated spaces within endothelium-lined cavities, and most endothelial cysts are lymphangiomatous.^[8]^ Histologically, the cystic channels were lined by endothelial cells, which were found intimately intermingled with the pancreatic parenchyma. The epithelial cells that make up the cavities can show specific staining for vascular cell markers, such as CD31 and CD34. In our review, we found that the endothelial cells lining the cystic spaces were strongly and diffusely positive for factor VIII-R Ag in 7 cases and positive for CD31 in 10 cases. CD34 was positive in 1 case (Table 3). D2-40 is the most recently identified marker of lymphatic endothelial cell differentiation and, is only expressed in lymphatic endothelial cells.^[12]^ And a total of 8 patients in our review showed positive results for D2-40 expression. Thus, the combination of multiple IHC markers including CD-31, CD-34, VIII-R, and D2-40, may help improve the diagnosis rate of pancreatic lymphangioma.

**Table 3:**
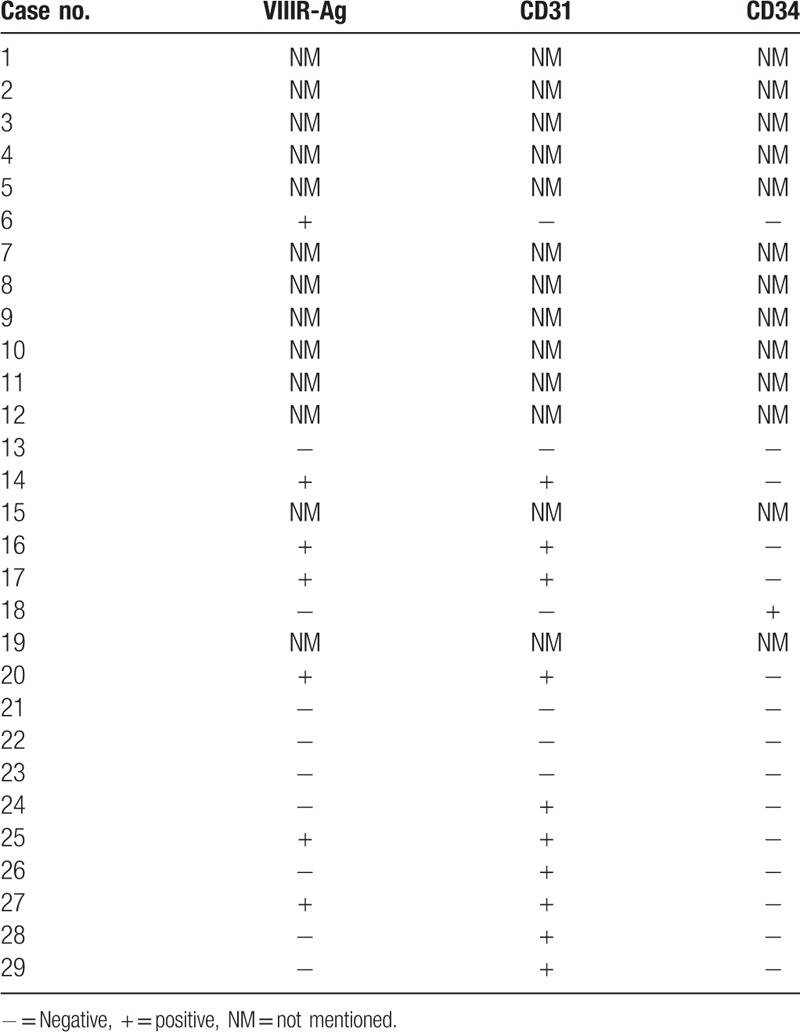
Immunohistochemical markers of the pancreatic lymphangioma specimens in the literature review.

### 3.4 Treatments for pancreatic lymphangioma

Surgery is the preferred treatment option for pancreatic lymphangioma, and incomplete excision leads to tumor recurrence.^[12]^ Based on the morphology and size of the tumor, different surgical options, including a simple excision of the mass or a pancreatic resection, such as Whipple procedure or distal pancreatectomy, must be considered.^[13]^ Although lymphangiomas are benign tumors, some studies suggest that the lymphangioma can encroach on adjacent organs and grow to an enormous size and that, resection of these invaded organs may be required for a complete excision.^[8]^

### 3.5 A new diagnostic strategy for pancreatic lymphangioma

As observed in the cases reported previously, the detection of cystic fluid contents may be necessary to diagnose of an abdominal cystic lymphangioma. Based on the histopathology of lymphangioma, some experts hold the view that the cystic fluid mostly originates from lymph-vessels, and lymphoid tissue may be found in the cystic fluid. Therefore, we aimed to apply US-guided puncture drainage combined with cell morphological examination to identify the origin of the tumor. In this case, we gathered the cystic fluid by US-guided fine needle aspiration. Through the cell morphological examination and IHC staining, we found that the cast-off cells mostly were lymphocytes and the cells were positive for IHC CD31 and D2-40. The use of such methods may be beneficial to diagnose abdominal cystic lymphangioma.

## Author contributions

Diyu Chen and Jian Wu designed the report; Zhen Lv, Xiaofeng Xu, and Chaofeng Ding performed the operation; Diyu Chen and Xiaode Feng wrote the manuscript; Jian Wu and Xiaode Feng reviewed and analyzed the data of literature; Zhen Lv provided advice during the preparation of the manuscript; Jian Wu made the critical revision.

**Conceptualization:** Jian Wu.

**Data curation:** Diyu Chen, Xiaode Feng, Xiaofeng Xu, Jian Wu.

**Formal analysis:** Diyu Chen.

**Resources:** Diyu Chen.

**Writing – original draft:** Diyu Chen, Xiaode Feng, Xiaofeng Xu, Jian Wu.

**Writing – review & editing:** Diyu Chen, Zhen Lv, Xiaofeng Xu, Chaofeng Ding, Jian Wu.
